# Correction: Cano-Vicent et al. Biocompatible Alginate Hydrogel Film Containing Acetic Acid Manifests Broad-Spectrum Antiviral and Anticancer Activities. *Biomedicines* 2023, *11*, 2549

**DOI:** 10.3390/biomedicines12030499

**Published:** 2024-02-23

**Authors:** Alba Cano-Vicent, Alberto Tuñón-Molina, Hamid Bakshi, Iman M. Alfagih, Murtaza M. Tambuwala, Ángel Serrano-Aroca

**Affiliations:** 1 Biomaterials and Bioengineering Lab, Centro de Investigación Traslacional San Alberto Magno, Universidad Católica de Valencia San Vicente Mártir, 46001 Valencia, Spain; alba.cano@mail.ucv.es (A.C.-V.); alberto.tunon@ucv.es (A.T.-M.); 2 Hormel Institute, University of Minnesota, Austin, MN 55912, USA; hamid.bakshi@gmail.com; 3 Department of Pharmaceutics, College of Pharmacy, King Saud University, Riyadh 4545, Saudi Arabia; fagih@ksu.edu.sa; 4 Brayford Pool Campus, Lincoln Medical School, University of Lincoln, Lincoln LN6 7TS, UK

## Error in Figure

In the original publication [1], there was a mistake in Figure 1 as published. Figure 1 had a human error. When we made the Figure, an image was taken as a reference and there was confusion with the photos. We have corrected this error. The corrected Figure 1 appears below.

Moreover, there was a mistake in Figure 8 as published. In Figure 8, the images that represent the control at 24 h and the control at 30 min are the same. This was a human error. We have corrected this error and we have changed the image that corresponds to the 24 h control. The corrected Figure 8 appears below.

The authors state that the scientific conclusions are unaffected. This correction was approved by the Academic Editor. The original publication has also been updated.

## Figures and Tables

**Figure 1:**
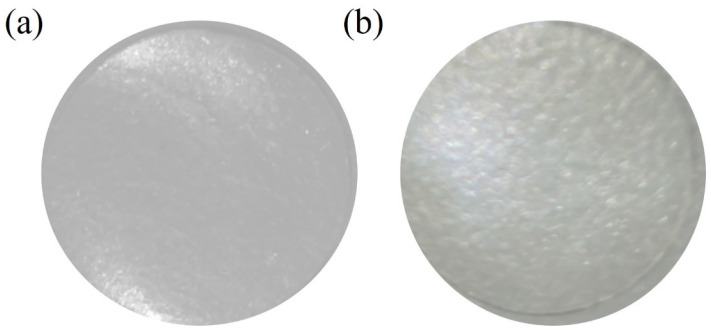
Macroscopic images of calcium alginate film containing acetic acid (**a**) and alginate film crosslinked with calcium (control film) (**b**).

**Figure 8:**
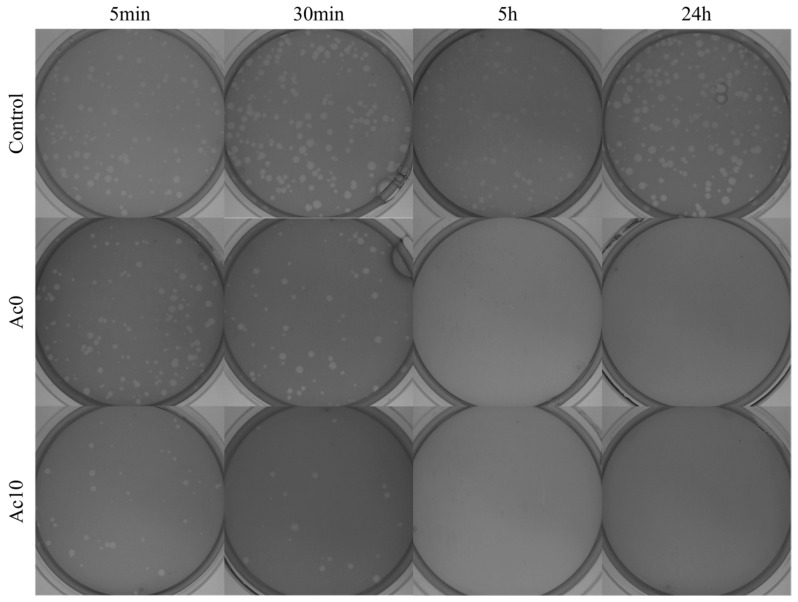
Loss of bacteriophage phi 6 viability measured by the double-layer method. Bacteriophage phi 6 titration images of undiluted samples for control, untreated film (Ac0), and film treated by acetic acid (Ac10) after 5 min, 30 min, 5 h and 24 h of viral contact.
